# Influenza vaccination and cardiovascular and respiratory outcomes in high-risk populations: an umbrella review of systematic reviews and meta-analyzes

**DOI:** 10.3389/fimmu.2026.1798398

**Published:** 2026-05-26

**Authors:** Chengyan Jin, Yan Zhang, Bin Wang, Peiyan Hua

**Affiliations:** Department of Thoracic Surgery, Second Hospital of Jilin University, Changchun, China

**Keywords:** cardiovascular disease, influenza vaccine, respiratory outcomes, secondary prevention, umbrella review

## Abstract

**Background:**

Influenza infection is an important trigger of acute cardiovascular events and respiratory decompensation in vulnerable populations. Although influenza vaccination may reduce cardiopulmonary morbidity and mortality, the overall certainty and methodological reliability of the evidence remain unclear.

**Objective:**

To synthesize and critically evaluate published systematic reviews and meta-analyses on the effectiveness and safety of influenza vaccination in populations at high risk of cardiovascular and respiratory complications.

**Methods:**

We conducted an umbrella review of systematic reviews and meta-analyses identified through PubMed, Embase, and the Cochrane Library from inception to December 1, 2025. Methodological quality was assessed using AMSTAR-2, evidence certainty using GRADE, and overlap of primary studies using citation matrices and corrected covered area (CCA). Given heterogeneity and review overlap, we performed a narrative synthesis and applied predefined rules to prioritize representative reviews.

**Results:**

Fourteen systematic reviews and meta-analyses were included. The strongest evidence supported an association between influenza vaccination and reduced major cardiovascular risk, particularly major adverse cardiovascular events in patients with ischemic heart disease and acute coronary syndrome. Vaccination was also associated with reduced cardiovascular mortality and all-cause mortality in some high-risk populations. In respiratory high-risk populations, vaccination was associated with fewer COPD exacerbations and lower laboratory-confirmed influenza incidence among older adults. Across an evidence base covering more than 230 million participants, no clear increase in serious adverse events was observed. The highest-certainty evidence was concentrated in selected cardiovascular and influenza-related outcomes, whereas stroke and several respiratory outcomes remained moderate to low in certainty because of heterogeneity and mixed study designs.

**Conclusion:**

Influenza vaccination appears to be a safe and clinically meaningful preventive strategy in populations at high risk of cardiopulmonary complications. The strongest current evidence supports selected cardiovascular endpoints and influenza-related outcomes. This umbrella review provides an integrated synthesis of cardiovascular, respiratory, and safety outcomes while explicitly addressing review quality, overlap of primary studies, and certainty of evidence.

**Systematic Review Registration:**

https://www.crd.york.ac.uk/prospero/, identifier CRD420251267371.

## Graphical Abstract

**Figure:**
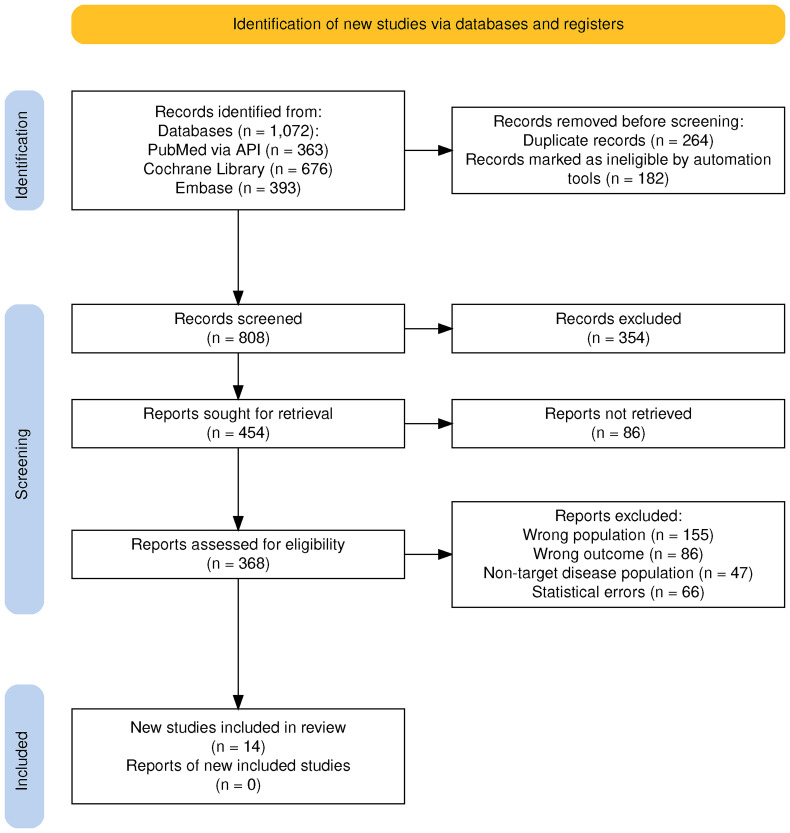

## Introduction

Seasonal influenza is one of the major sources of the global burden of respiratory diseases. In addition, seasonal influenza is increasingly recognized as a major cause of acute cardiovascular events. Beyond causing primary respiratory complications such as pneumonia, there is growing evidence that influenza infection may trigger a ‘flu–myocardial infarction cascade,’ thereby becoming an important clinical trigger for sudden cardiovascular instability (1, 2). Epidemiological studies show a significant temporal correlation between the timing of influenza outbreaks and cardiovascular incidence and mortality (1). These correlations also include increased hospitalizations and deaths associated with acute myocardial infarction. An important self-controlled case series study found that within the first 7 days after laboratory-confirmed influenza, patients had a significantly increased risk of acute myocardial infarction, especially among older adults and those with atherosclerosis (3).

This association is also biologically plausible. Influenza infection can trigger a strong systemic inflammatory response, manifested by increases in interleukin-1β, interleukin-6, and tumor necrosis factor-α. This suggests that acute cardiovascular events may be associated with peaks in inflammation. First, due to inflammation-induced sympathetic activation, heart rate increases and peripheral blood vessels constrict, thereby increasing myocardial oxygen consumption. Second, inflammatory mediators can alter matrix metalloproteinases in atherosclerotic plaques, reducing the stability of the fibrous cap and making plaques more prone to rupture. Third, influenza infection promotes thrombosis by increasing platelet activation and fibrinogen levels. Endothelial activation and immune-thrombotic interactions may also contribute to vascular instability during acute infection. Based on these converging mechanisms, influenza vaccination may reduce downstream cardiopulmonary complications by preventing infection and attenuating subsequent inflammatory and thrombotic cascades.

On the basis of these epidemiological and mechanistic findings, major professional societies such as the American Heart Association (AHA) and the European Society of Cardiology (ESC) recommend that high-risk cardiovascular patients receive an annual influenza vaccination. However, these high-risk individuals still have not been vaccinated, especially in routine cardiovascular clinical practice, where the coverage level needed for prevention is far from being achieved. Limitations in the existing evidence base may account for this gap between evidence and practice. Since most early studies were observational, they are prone to unnecessary confounding effects, such as the ‘healthy vaccinee bias,’ where individuals who are generally healthier are more likely to be vaccinated, thereby overestimating the benefits of the vaccine. In addition, because large, high-quality randomized controlled trials were rarely conducted in the past, clinicians have lacked sufficiently robust randomized evidence to determine the extent to which influenza vaccination improves cardiovascular outcomes.

However, in recent years, the evidence landscape has undergone significant changes. With the publication of the Influenza Vaccination After Myocardial Infarction (IAMI) trial and updated meta-analyses of randomized data by Liu et al. (2025) and Veroniki et al. (2024), the evidence supporting an association between influenza vaccination and a reduced risk of adverse cardiovascular outcomes in patients with ischemic heart disease and acute coronary syndrome has strengthened substantially (4, 5). By using larger sample sizes, more optimized trial designs, and more extensive follow-ups, these new studies have partially filled the long-term evidence gaps in previous cardiovascular prevention research. In addition, the number of studies on vaccine safety, vulnerable populations, and respiratory outcomes is rapidly increasing, making the overall evidence base more complex, and there may be overlap between different reviews.

With the increase in high-quality evidence, it has become necessary to conduct a comprehensive re-evaluation of the existing literature to determine the consistency, certainty, and clinical significance of evidence across different outcomes and populations. Against this backdrop, we conducted an umbrella review, including systematic reviews and meta-analyses published up to 2025, and used established evidence evaluation frameworks such as AMSTAR-2 and GRADE to systematically assess methodological quality and evidence certainty (6). Compared with previous reviews, this study aims to provide a higher-level synthesis of cardiovascular, respiratory, and safety outcomes in high-risk populations. It also explicitly addresses the issue of overlapping evidence among different reviews and further clarifies the reliability and strength of the existing evidence base.

## Methods

### Study design and protocol registration

This study is an umbrella review that includes and comprehensively evaluates systematic reviews and meta-analyses related to influenza vaccination and cardiovascular, respiratory, and safety outcomes in high-risk populations. The study design and reporting follow the Preferred Reporting Items for Systematic Reviews and Meta-Analyses (PRISMA) framework and are conducted with reference to current methodological recommendations for umbrella reviews (7). The study protocol has been pre-registered in PROSPERO (CRD420251267371).

### Inclusion and exclusion criteria

We include systematic reviews that assess the effects of influenza vaccines in populations at high-risk of cardiovascular or respiratory complications, whether or not they include meta-analyses. The target populations include, but are not limited to, individuals with cardiovascular disease (CVD), ischemic heart disease (IHD), acute coronary syndrome (ACS), chronic obstructive pulmonary disease (COPD), older adults, and other clinically vulnerable populations. Included studies must report at least one of the following outcomes: cardiovascular outcomes, respiratory outcomes, hospitalization, death, influenza-related outcomes, or vaccine safety outcomes.

The inclusion criteria are as follows:

1. Clearly describe a systematic search strategy;
2. Focus on influenza vaccine as the main exposure factor or intervention;
3. Report outcomes related to cardiopulmonary risk, influenza-related morbidity, or vaccine safety;
4. Published in English in a peer-reviewed journal.

Narrative reviews, editorials, commentaries, conference abstracts without full-text, and studies that do not focus on influenza vaccine or are unrelated to the predefined target population and outcomes are excluded.

### Search strategy

From the establishment of the database until December 1, 2025, we conducted systematic searches in PubMed, Embase, and the Cochrane Library. The search terms combined subject headings and free text, covering concepts such as influenza vaccines, cardiovascular diseases, respiratory diseases, high-risk/vulnerable populations, and systematic reviews/meta-analyses. The complete electronic search strategies for each database are provided in **Supplementary Appendix 1**.

In short, the PubMed search strategy combines influenza vaccine–related terms (such as “Influenza Vaccines”[Mesh], “influenza vaccine”, “flu vaccine”, and “influenza vaccination”) with terms for cardiovascular and respiratory diseases (such as “Cardiovascular Diseases”[Mesh], “Myocardial Infarction”[Mesh], “Stroke”[Mesh], “Respiratory Diseases”[Mesh], COPD, asthma), high-risk population terms (such as older adults, chronic disease, comorbidity), and review-level evidence terms (such as “Meta-Analysis” and “Systematic Review”). Embase and Cochrane Library used corresponding database-specific adjusted versions.

### Literature screening process

The literature screening included five stages: database retrieval, deduplication, title/abstract screening, full-text eligibility assessment, and final inclusion. Two researchers independently evaluated potential eligible studies, and any disagreements were resolved through discussion, with senior researchers adjudicating when necessary.

To improve the efficiency of the initial PubMed screening stage, we used an automated-assisted process under full manual supervision. This system integrates PubMed Entrez application programming interface (API) metadata retrieval, a full-text-priority retrieval module, and a structured large language model (LLM) to assist in relevance assessment. The system architecture and detailed process are provided in **Supplementary Appendix 2**.

In short, when the full-text is accessible, the system prioritizes a hierarchical full-text retrieval strategy: it first attempts to obtain Journal Article Tag Suite XML (JATS XML) based on PubMed Central identifier (PMCID), then tries to retrieve the web full-text based on digital object identifier (DOI), and if the full-text cannot be obtained, it falls back to abstract evaluation in a non-strict full-text mode. Subsequently, the system uses structured prompts containing the title, abstract, and available full-text paragraphs to assess the relevance of each record. The LLM output is in a structured JSON format, including whether the topic matches, confidence score, judgment rationale, and supporting citations. To reduce model variability, each literature piece undergoes five independent rounds of inference, and the result with the highest confidence is selected for researchers’ reference. Automated tools were used only for early relevance assessment and prioritization; all final inclusion and exclusion decisions were made by human reviewers. Records marked as “potentially relevant” undergo manual full-text review, and exclusion results with uncertainty are also rechecked.

### Data extraction

For each included review, we extracted the following information: first author, year of publication, target population, outcome domains, types of study designs included in the original research, number of studies included, total sample size, pooled effect sizes and their 95% confidence intervals, heterogeneity statistics, and methodological quality ratings. When applicable, information related to overlap assessment, certainty of evidence, and safety outcomes was also extracted. Data extraction was performed independently by two reviewers and cross-checked to ensure accuracy. All analyzes and data handling were performed using R version 4.3.3.

### Methodological quality assessment

The methodological quality of systematic reviews and meta-analyses was assessed using AMSTAR-2 (A MeaSurement Tool to Assess Systematic Reviews 2). Each review was categorized into one of four overall levels: high, moderate, low, or critically low. To enhance the transparency of quality assessment, we presented the distribution of quality at both the item level and the overall review level in the supplementary figures.

### Assessment of evidence certainty

The certainty of evidence for the main outcomes was assessed using the GRADE (Grading of Recommendations Assessment, Development and Evaluation) approach. Based on dimensions such as risk of bias, inconsistency, indirectness, imprecision, and publication-related issues, the evidence was categorized as high, moderate, low, or very low certainty. We used GRADE to provide supportive interpretation for the most clinically relevant umbrella-level findings, especially when there was high heterogeneity or low methodological quality of the reviews, to avoid overinterpretation. A summary of the certainty of evidence is shown in **Supplementary Table 2**.

### Handling of overlapping reviews and primary studies

Since umbrella reviews often include multiple systematic reviews addressing the same or similar outcomes, we explicitly assessed the overlap of primary studies among the included reviews. The overlap was evaluated using a citation matrix and the CCA, and separate matrices were constructed for cardiovascular outcomes, respiratory/vulnerable population outcomes, and safety/mixed clinical outcomes. To avoid double-counting the same underlying evidence in interpretation, we did not perform new quantitative meta-analyses across different reviews. Instead, for outcome areas with obvious conceptual overlap, we selected representative reviews as the primary basis for interpretation according to predefined priority rules. The priority rules included: recency of search time, target population match, focus on randomized controlled trials, methodological quality, outcome specificity, and sample size. The framework for selecting representative reviews is shown in **Supplementary Table 1**. Other overlapping reviews were retained for consistency comparison and to provide background explanations for differences in populations, study designs, and effect estimates.

### Data integration

Given the anticipated heterogeneity among the included reviews in terms of study populations, original study designs, outcome definitions, and overlap of underlying primary studies, we employed a narrative synthesis rather than conducting a new umbrella-level meta-analysis. The synthesis framework revolves around three main areas: cardiovascular protection, respiratory/vulnerable population outcomes, and safety. Within each area, we prioritized giving greater interpretive weight to the most recent, methodologically reliable reviews, primarily based on randomized evidence, while incorporating AMSTAR-2 ratings, GRADE certainty, heterogeneity, and overlap into the final interpretation (8, 9). This strategy aims to provide a more transparent and clinically meaningful summary of the current evidence base, while minimizing the interference of duplicate evidence and low-quality reviews.

The LLM-assisted component was used strictly for preliminary prioritization and did not influence final eligibility decisions, which were determined solely by independent human reviewers following predefined criteria.

## Results

All 14 included reviews were retained in the umbrella evidence base; for overlapping outcomes, representative reviews were prioritized for the main-text interpretation, whereas the remaining reviews were used for consistency comparison and are summarized in **Table 1**; **Supplementary Table 1**, and the corresponding figure legends (4, 5, 8–19).

**Table 1:** Summary characteristics of included systematic reviews and meta-analyses.

Author (Year)	Population	Outcome	Design	k	N (total)	Metric^a^	ES (95% CI)	I² (%)^b^	AMSTAR-2^c^
1. CVD protection									
Liu (4)	IHD Patients	MACE	RCTs	5	4,656	RR	0.67 (0.52-0.87)	36	Low
Liu (4)	IHD Patients	All-cause Mortality	RCTs	5	4,656	RR	0.58 (0.40-0.84)	0	Low
Zangiabadian (8)	CVD Patients	Composite CV Events	RCTs	6	7,358	RR	0.55 (0.41-0.73)	50	Moderate
Zangiabadian (8)	High-risk Adults	Major CV Events	Cohort	5	206,355	OR	0.89 (0.77-1.04)	88	Moderate
Zangiabadian (8)	Adults (≥40/65y)	Major CV Events	Case-Control	6	243,228	OR	0.70 (0.57-0.86)	98	Moderate
Liu (16)	CVD Patients	CV Mortality	RCTs	3	8,358	RR	0.80 (0.60-1.07)	37	High
Liu (16)	Adults + CVD	All-cause Hosp.	RCTs	2	8,265	RR	0.86 (0.76-0.97)	0	High
Behrouzi (10)	Recent ACS	MACE	RCTs	6	4,324	RR	0.55 (0.41-0.75)	42	Critically low
Behrouzi (10)	Recent ACS	CV Mortality	RCTs	3	2,729	RR	0.44 (0.23-0.85)	0	Critically low
Gupta (13)	Heart Failure	All-cause Mortality	Obs	6	247,842	RR	0.75 (0.71-0.79)	72	Low
Rodrigues (17)	Prev. Stroke	Recurrent Stroke	Obs	3	NR	RR	0.75 (0.70-1.01)	NR	Critically low
Yedlapati (18)	CVD Patients	MACE	Mixed^d^	16	237,058	RR	0.87 (0.80-0.94)	78	Low
Zahhar (2024)	General/At-risk	Stroke Incidence	Mixed^d^	44	>200M^e^	OR	0.81 (0.77-0.86)	98	High
Zahhar (2024)	Stroke Patients	All-cause Mortality	Obs	4	77,508	OR	0.50 (0.37-0.68)	72	High
Veroniki (5)	Older Adults	All-cause Mortality	RCTs	20	140,577	OR	1.01 (0.23-4.49)	8	High
2. Respiratory/vulnerable populations									
Bao (9)	COPD Patients	Acute Exacerbation	Mixed^d^	10	17,972	RR	0.37 (0.21-0.61)	98	Low
Kopsaftis (15)	COPD Patients	Hospital Admission	RCTs	6	1,627	RR	0.37 (0.10-1.35)	48	Moderate
Cheng (11)	COPD Patients	Exacerbation/Hosp	Obs	3	~80k^e^	RR	0.82 (0.47-1.43)	88	Moderate
Veroniki (5)	Older Adults	Lab-confirmed Flu	RCTs	9	52,202	OR	0.23 (0.11-0.51)	0	High
Veroniki (5)	Older Adults	ILI	RCTs	2	854	OR	0.39 (0.15-1.02)	0	High
Ferdinands (12)	Older Adults	Flu-assoc. Hosp	RCTs	5	~40k^e^	RR	0.75 (0.49-1.00)	0	High
Jarvis (14)	Infants (<6m)	Lab-confirmed Flu	RCTs	4	9,336	RR	0.66 (0.50-0.85)	34	Low
3. Safety									
Veroniki (5)	Older Adults	Vascular AEs (HD vs SD)	RCTs	4	~50k^e^	IRR	0.69 (0.49-0.97)	97	High
Veroniki (5)	Older Adults	Vascular AEs (Adj vs SD)	RCTs	2	~10k^e^	IRR	0.90 (0.54-1.50)	NR	High
Liu (16)	CVD Patients	Serious AEs (SAE)	RCTs	2	3,229	RR	1.14 (0.73-1.77)	2	High
Liu (16)	CVD Patients	Any Adverse Events	RCTs	2	3,229	RR	1.13 (0.73-1.76)	1	High

This table provides an overview of all included systematic reviews and meta-analyses evaluating the association between influenza vaccination and cardiovascular, respiratory, and safety outcomes across diverse populations. Extracted characteristics include population type, outcome definition, study design, number of included studies (k), total sample size (N), effect size metric, pooled effect estimates with 95% confidence intervals (CIs), heterogeneity (I²), and methodological quality assessed using the AMSTAR-2 tool.

ACS, acute coronary syndrome; AE, adverse event; Adj, adjuvanted vaccine; CVD, cardiovascular disease; CV, cardiovascular; HD, high-dose vaccine; IHD, ischemic heart disease; ILI, influenza-like illness; IRR, incidence rate ratio; MACE, major adverse cardiovascular events; NR, not reported; Obs, observational; RCTs, randomized controlled trials; SD, standard-dose vaccine.

a. Effect size metrics reflect pooled estimates directly extracted from the included systematic reviews or meta-analyses. Metrics include risk ratio (RR), odds ratio (OR), and incidence rate ratio (IRR), depending on the original analytic framework.

b. Heterogeneity was reported using I² statistics as provided by the original publications. “NR” indicates that heterogeneity values were not reported.

c. AMSTAR-2 ratings denote the overall methodological quality of each review (High, Moderate, Low, Critically low) based on 16 appraisal domains.

d. Mixed-design reviews include combinations of RCTs, cohort studies, case-control studies, or registry-based analyzes.

e. Sample sizes (N) reflect total pooled populations as reported in each meta-analysis; values such as “~40k” or “>200M” represent approximations provided in the source publications.

### Study selection and characteristics of included reviews

**Figure 1:**
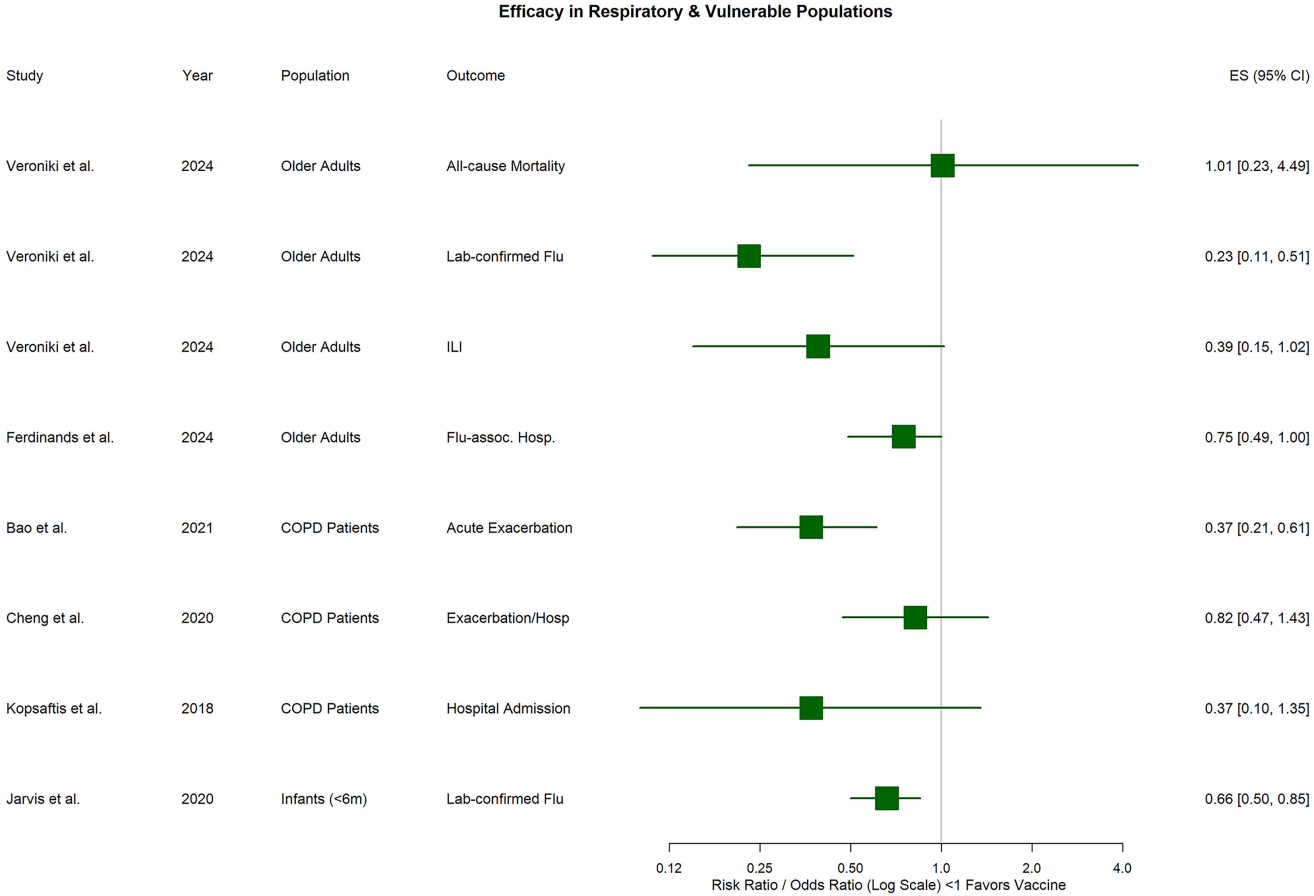
Efficacy of influenza vaccination on cardiovascular and stroke outcomes (2020–2025). This figure summarizes pooled effect estimates from recent systematic reviews and meta-analyses evaluating the association between influenza vaccination and major cardiovascular outcomes, including MACE, all-cause mortality, cardiovascular mortality, stroke incidence, and recurrent stroke. Effect estimates (risk ratios or odds ratios) and 95% confidence intervals are displayed using a log-scale forest plot. Data sources include meta-analyses of randomized controlled trials (Liu et al., 2025) and large-scale observational syntheses (Zahhar et al., 2024; Behrouzi et al., 2022; Gupta et al., 2022; Yedlapati et al., 2021; Zangiabadian et al., 2020; Rodrigues et al., 2021). Overall, pooled estimates generally favored risk reduction, with the strongest support observed for selected cardiovascular outcomes.

The literature search identified 1,072 records through database searching. After removal of duplicates, an automation-supported prescreening process excluded 284 clearly irrelevant PubMed records, and manual dual screening of EMBASE and the Cochrane Library excluded a further 420 records. Following full-text assessment, 14 systematic reviews, meta-analyses, or umbrella reviews published between 2018 and 2025 met the eligibility criteria and were included in this umbrella review. The study selection process is shown in the PRISMA 2020 flow diagram (**Figure 1**), and the characteristics of the included reviews are summarized in **Table 1**.

The included reviews covered a broad range of high-risk populations, including patients with CVD, IHD, ACS, heart failure, previous stroke, COPD, and older adults. The underlying primary studies included randomized controlled trials (RCTs), observational studies, or both. Outcome domains included major adverse cardiovascular events (MACE), cardiovascular mortality, all-cause mortality, hospitalization, stroke incidence, recurrent stroke, COPD exacerbation, laboratory-confirmed influenza, influenza-like illness (ILI), and vaccine safety outcomes.

### Methodological quality of included reviews

Methodological quality varied substantially across the included reviews. Based on AMSTAR-2, 4 reviews were rated as high quality, 3 as moderate quality, 5 as low quality, and 2 as critically low quality. Higher-quality reviews generally reported preregistered protocols, comprehensive search strategies, and formal risk-of-bias assessment, whereas lower-quality reviews more often lacked prospective registration, detailed bias appraisal, or transparent reporting of analytic decisions. In the umbrella-level interpretation, greater weight was therefore assigned to more recent reviews, reviews with higher AMSTAR-2 ratings, and reviews emphasizing randomized evidence. Detailed AMSTAR-2 results are provided in **Supplementary Figures 1**-**3**.

### Overlap assessment and selection of representative reviews

Because several reviews addressed overlapping populations and outcomes, we formally evaluated primary-study overlap using citation matrices and CCA. Overlap was low overall but differed across domains: CCA was 1.13% for cardiovascular outcomes, 4.55% for respiratory and vulnerable-population outcomes, and 9.72% for safety and composite outcomes, corresponding to no overlap, slight overlap, and moderate overlap, respectively. To avoid overemphasizing duplicated evidence, we did not perform a new umbrella-level meta-analysis across overlapping reviews. Instead, representative reviews were selected *a priori* on the basis of recency, methodological quality, population specificity, emphasis on RCT evidence, outcome specificity, and sample size. **Supplementary Table 1** summarizes the representative-review selection strategy across overlapping outcomes. Citation matrices illustrating overlap of primary studies across reviews are provided in **Supplementary Figures 4**-**6**, together with the corresponding CCA estimates.

### Cardiovascular outcomes

The most consistent and clinically relevant finding in this umbrella review was an association between influenza vaccination and reduced cardiovascular risk in high-risk populations. The strongest evidence was observed for MACE in patients with IHD and recent ACS. In the most up-to-date RCT-based meta-analysis, influenza vaccination was associated with a 33% relative reduction in MACE among patients with IHD (RR 0.67, 95% CI 0.52-0.87; 5 RCTs, n=4,656; I²=36%), and this outcome was graded as high-certainty evidence. A stronger effect was observed in patients with recent ACS, in whom vaccination was associated with a 45% relative reduction in MACE (RR 0.55, 95% CI 0.41-0.75; 6 RCTs, n=4,324; I²=42%). Other overlapping reviews, including Behrouzi et al. (2022), Yedlapati et al. (2021), Gupta et al. (2022), and Rodrigues et al. (2021), were retained for consistency comparison and showed directionally similar findings across related cardiovascular endpoints (10, 13, 17, 18).

Protective associations were also observed for mortality outcomes, although certainty varied by endpoint. Among patients with IHD, influenza vaccination was associated with lower all-cause mortality (RR 0.58, 95% CI 0.40-0.84; 5 RCTs, n=4,656; I²=0%). For cardiovascular mortality in broader CVD populations, the pooled estimate also favored vaccination, although the confidence interval crossed the null (RR 0.80, 95% CI 0.60-1.07; 3 RCTs, n=8,358; I²=37%), corresponding to moderate-certainty evidence because of imprecision. In patients with recent ACS, cardiovascular mortality was also reduced in one RCT-based synthesis (RR 0.44, 95% CI 0.23-0.85; 3 RCTs, n=2,729; I²=0%).

**Figure 2:**
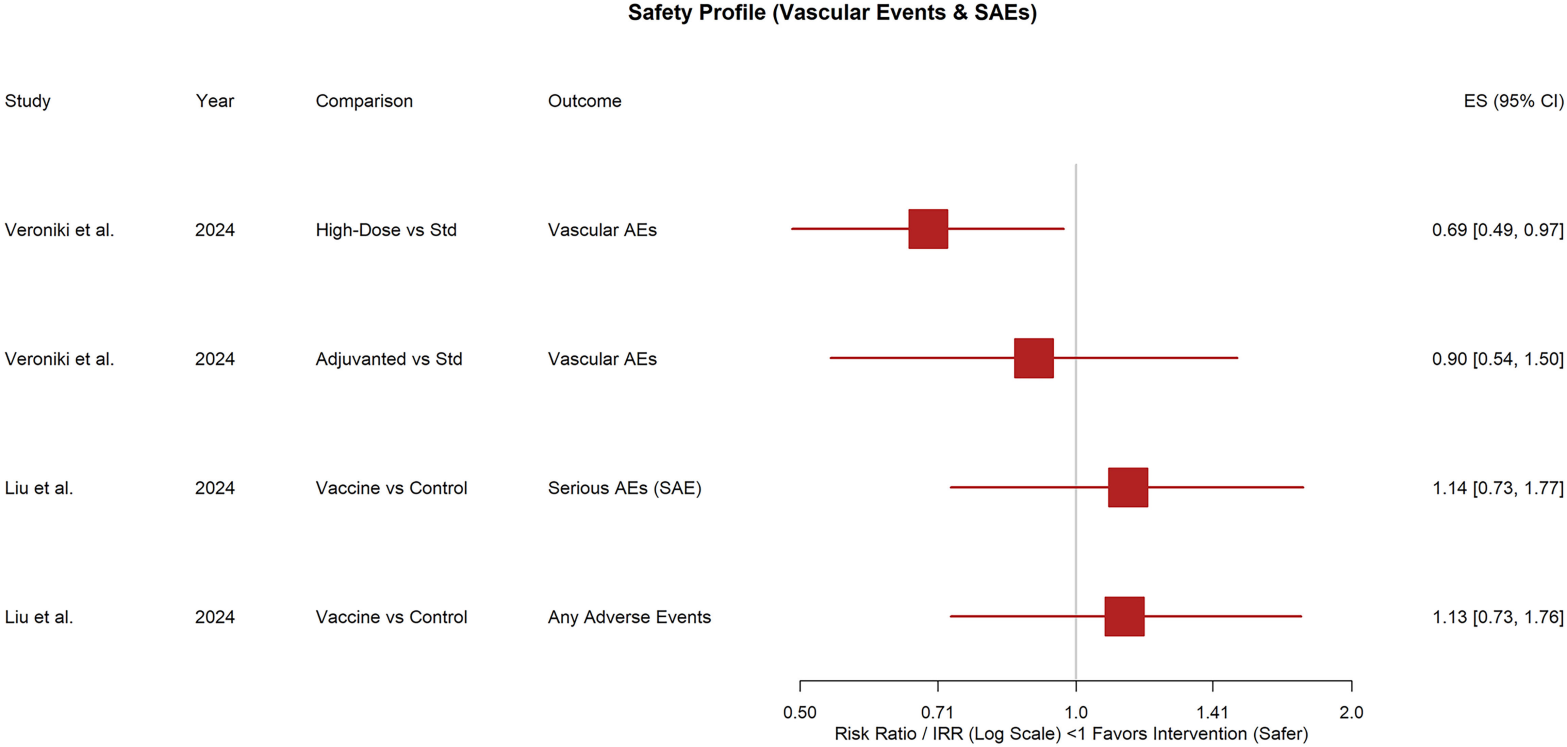
Efficacy of influenza vaccination in respiratory and other vulnerable populations. This figure presents pooled effect estimates for respiratory outcomes among older adults, patients with COPD, and other vulnerable populations. Outcomes include all-cause mortality, laboratory-confirmed influenza, influenza-like illness, COPD acute exacerbations, hospitalization, and influenza-associated hospitalization. Meta-analyses demonstrate substantial reductions in influenza-related outcomes among older adults (Veroniki et al., 2024) and significant reductions in COPD exacerbations and hospitalizations (Bao et al., 2021; Cheng et al., 2020; Kopsaftis et al., 2018). Effect estimates are presented as risk ratios or odds ratios with 95% confidence intervals on a log-transformed scale.

Other cardiovascular outcomes generally showed directionally favorable estimates, but with lower certainty. Stroke incidence was reduced in a large mixed-design synthesis including 44 studies and more than 200 million participants (OR 0.81, 95% CI 0.77-0.86), but heterogeneity was extreme (I²=98%), and GRADE certainty was low. Among stroke survivors, all-cause mortality was also lower in vaccinated individuals (OR 0.50, 95% CI 0.37-0.68; 4 studies, n=77,508; I²=72%). Recurrent stroke risk showed a favorable but imprecise association (RR 0.75, 95% CI 0.70-1.01; 3 observational studies). Overall, cardiovascular benefit was most convincingly supported for MACE in IHD and ACS, whereas findings for stroke-related outcomes were more limited by heterogeneity and mixed study designs. These cardiovascular findings are summarized in **Figure 2**.

### Respiratory and other vulnerable-population outcomes

Influenza vaccination also showed clinically relevant benefits in respiratory high-risk populations. Among patients with COPD, vaccination was associated with a marked reduction in acute exacerbations (RR 0.37, 95% CI 0.21-0.61; 10 studies, n=17,972), although between-study heterogeneity was high (I²=98%) and certainty was low. Additional COPD-related outcomes were directionally favorable but less precise, including hospital admission in RCTs (RR 0.37, 95% CI 0.10-1.35; 6 studies, n=1,627; I²=48%) and exacerbation or hospitalization in observational studies (RR 0.82, 95% CI 0.47-1.43; approximately 80,000 participants; I²=88%). Additional overlapping reviews, including Cheng et al. (2020), Kopsaftis et al. (2018), and Jarvis et al. (2020), were retained for contextual comparison across respiratory and other vulnerable populations (11, 14, 15).

**Figure 3:**
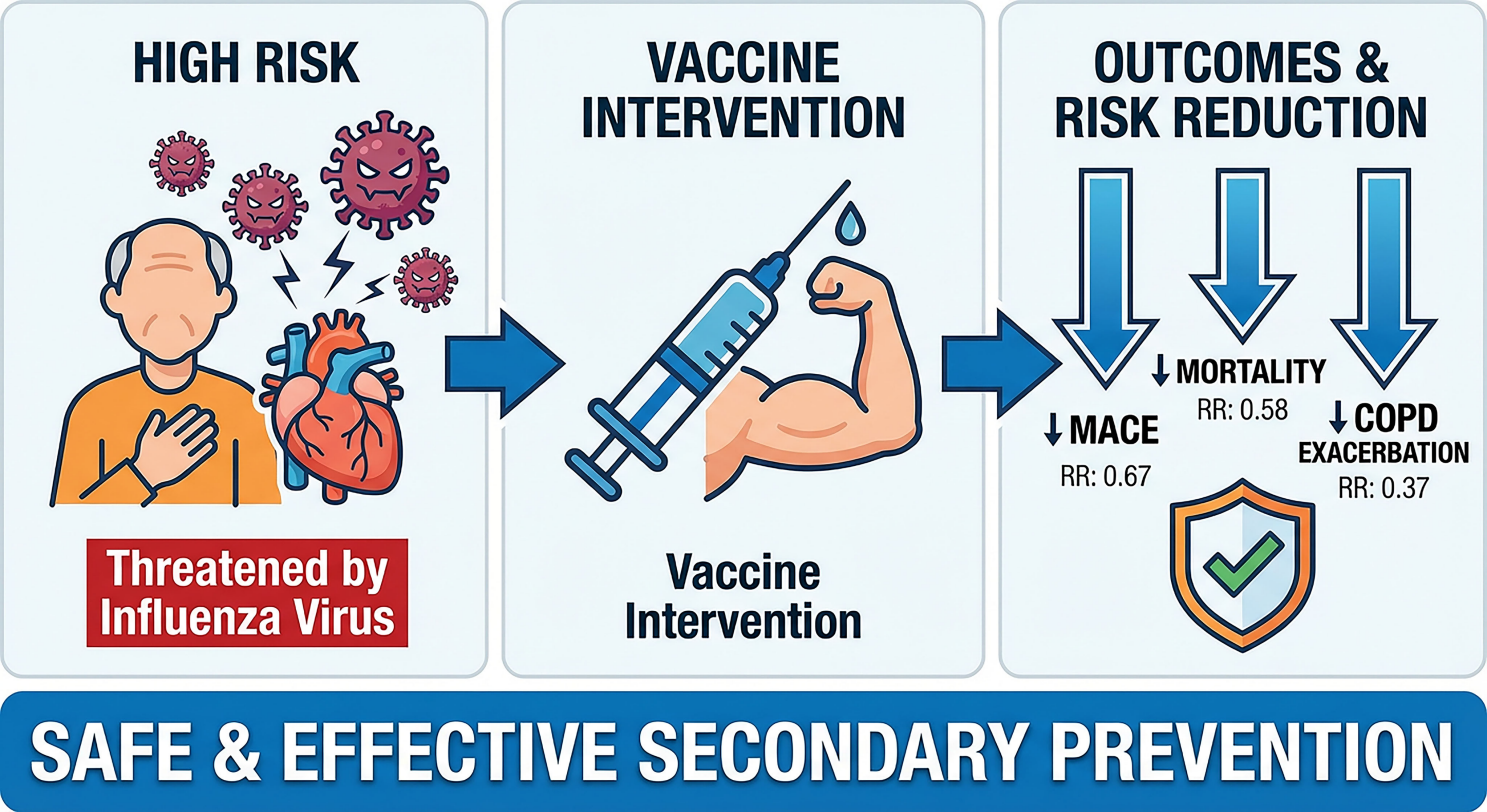
Safety profile of influenza vaccination (vascular events and serious adverse events). This figure summarizes safety outcomes from randomized controlled trials and comparative analyzes of influenza vaccines. Evaluated outcomes include vascular adverse events (high-dose vs. standard-dose and adjuvanted vs. standard-dose formulations), SAEs, and any adverse events. Across included studies (Veroniki et al., 2024; Liu et al., 2024), no clear increase in the risk of SAEs or overall adverse events was observed, and high-dose vaccines were associated with lower rates of vascular events. Effect sizes are reported as risk ratios or incidence rate ratios with 95% confidence intervals on a log scale.

In older adults, the strongest respiratory finding was for laboratory-confirmed influenza, for which vaccination was associated with a substantial reduction in risk (OR 0.23, 95% CI 0.11-0.51; 9 RCTs, n=52,202; I²=0%), representing high-certainty evidence. For influenza-like illness, the pooled estimate was also favorable but imprecise (OR 0.39, 95% CI 0.15-1.02; 2 RCTs, n=854; I²=0%), and certainty was low because of the small sample size and the confidence interval crossing the null. Influenza-associated hospitalization in older adults also showed a borderline favorable estimate (RR 0.75, 95% CI 0.49-1.00; 5 RCTs, approximately 40,000 participants; I²=0%). Taken together, these findings support clinically relevant respiratory protection, with the most robust evidence seen for prevention of laboratory-confirmed influenza in older adults and a favorable, though more heterogeneous, signal for COPD exacerbation reduction. These respiratory and vulnerable-population outcomes are summarized in **Figure 3**.

### Safety outcomes

Across the included evidence base, influenza vaccination was not associated with a clear increase in serious harm. In RCT-based analyzes of patients with CVD, there was no significant difference between vaccine and control groups for serious adverse events (RR 1.14, 95% CI 0.73-1.77; 2 RCTs, n=3,229; I²=2%) or for any adverse events (RR 1.13, 95% CI 0.73-1.76; 2 RCTs, n=3,229; I²=1%). These findings were graded as moderate certainty because the confidence intervals were wide and crossed 1.0, but they did not indicate a statistically significant increase in harm.

**Figure 4:**
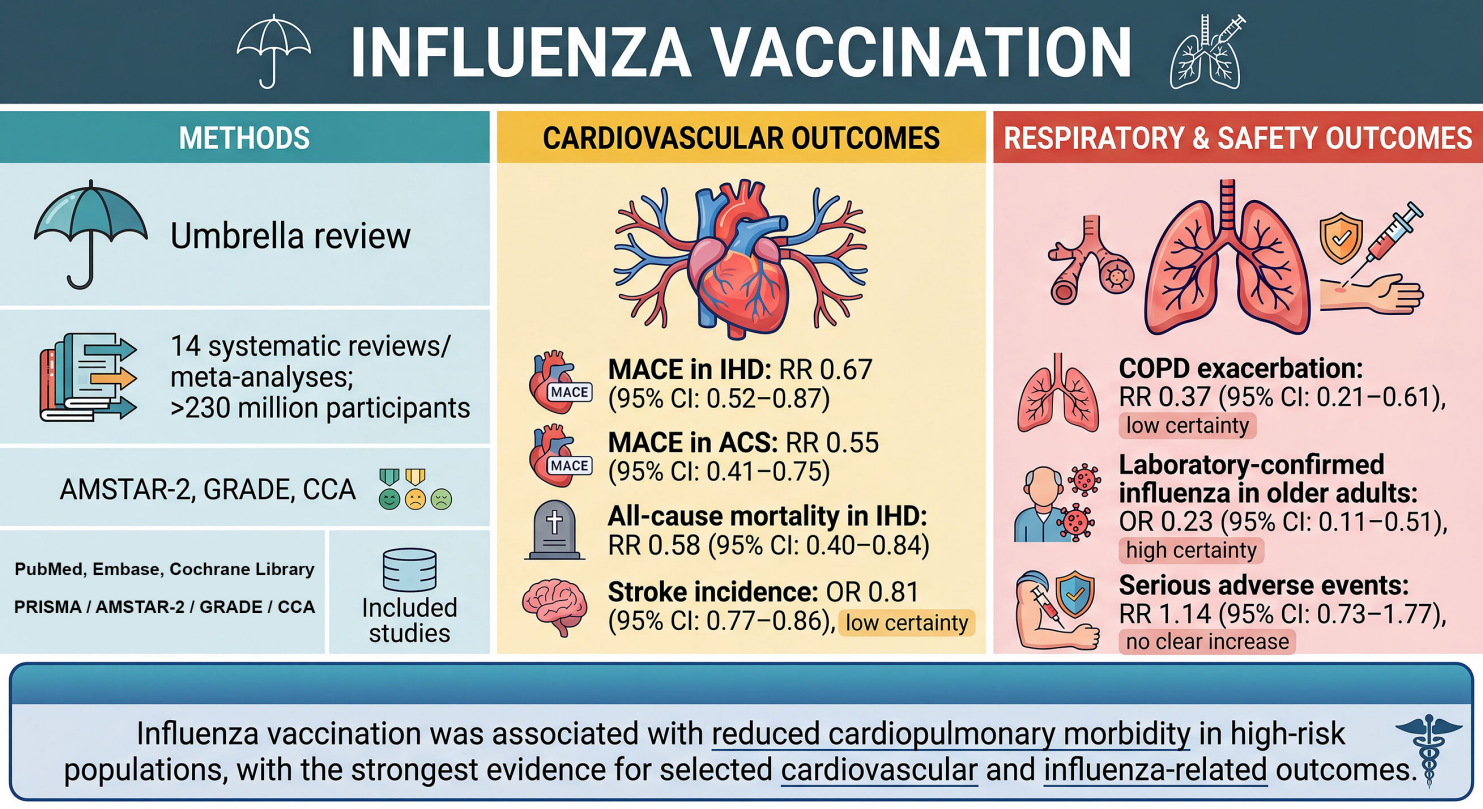
Proposed immunological and clinical mechanisms underlying the cardiopulmonary protective effects of influenza vaccination in high-risk populations. People at high-risk for cardiovascular and respiratory diseases are more susceptible to influenza, which can cause systemic inflammation, endothelial dysfunction, plaque instability, prothrombotic state, and acute respiratory deterioration. Influenza vaccination may induce a protective immune response, reduce viral infection and downstream inflammatory burden, thereby reducing the risk of MACE, all-cause mortality, and worsening of COPD. The relative risk reduction shown in the figure comes from the summary estimation of high-quality randomized and observational evidence synthesized in this review, supporting the idea that influenza vaccination is a safe and effective secondary prevention strategy for vulnerable populations.

Among older adults, high-dose influenza vaccines were associated with fewer vascular adverse events than standard-dose vaccines (IRR 0.69, 95% CI 0.49-0.97; 4 RCTs, approximately 50,000 participants), although heterogeneity was substantial (I²=97%) and certainty was low. Adjuvanted versus standard-dose formulations did not show a clear difference in vascular adverse events (IRR 0.90, 95% CI 0.54-1.50; 2 RCTs, approximately 10,000 participants). Overall, the available evidence supports a favorable safety profile for influenza vaccination in vulnerable populations, without a clear signal of increased serious adverse events. These safety outcomes are summarized in **Figure 4**.

### Certainty of evidence across outcomes

GRADE assessment showed that certainty was not uniform across outcome domains. The highest-certainty evidence was observed for MACE in IHD (RR 0.67, 95% CI 0.52-0.87) and laboratory-confirmed influenza in older adults (OR 0.23, 95% CI 0.11-0.51), both based on randomized evidence with low or absent heterogeneity. Cardiovascular mortality and serious adverse events were supported by moderate-certainty evidence, largely limited by imprecision. By contrast, stroke incidence, COPD exacerbation, ILI in older adults, and high-dose vascular adverse events were graded as low-certainty outcomes because of substantial heterogeneity, mixed study designs, or small sample sizes. Full GRADE judgments and reasons for downgrading are presented in **Supplementary Table 2**.

### Umbrella-level synthesis

Overall, this umbrella review supports three main conclusions. First, influenza vaccination is associated with reduced cardiovascular risk in selected high-risk populations, with the strongest evidence observed for MACE in patients with IHD and recent ACS, and additional supportive evidence for mortality reduction in some cardiovascular subgroups. Second, vaccination is also associated with respiratory benefit, particularly reduced COPD exacerbation and a marked reduction in laboratory-confirmed influenza among older adults. Third, current evidence does not indicate a clear increase in serious adverse events. Taken together, these findings support influenza vaccination as a potentially important preventive strategy in populations at high-risk of cardiopulmonary complications.

## Discussion

### Overall explanation and the contribution of this umbrella review

In this umbrella review, we synthesized the latest systematic reviews and meta-analyses on the effects of influenza vaccination in populations at high-risk of cardiovascular and respiratory complications. Three main findings emerged. First, the most consistent current evidence supports an association between influenza vaccination and a reduced risk of major cardiovascular outcomes, particularly MACE in populations with IHD and ACS; this evidence base has been significantly strengthened by recent RCTs and RCT-focused meta-analyses (3, 4, 8). Second, influenza vaccination is also associated with clinically relevant benefits in populations at high-risk for respiratory conditions and other vulnerable groups, including reductions in acute exacerbations of COPD and decreased incidence of laboratory-confirmed influenza in older adults (5, 9). Third, in the context of a very large body of accumulated evidence, no clear signal of increased serious adverse events (SAEs) has been observed, supporting that influenza vaccination has good safety even in clinically vulnerable populations (16).

An important contribution of this study is that it does not simply replicate previous meta-analyses focused on a single disease area; rather, it provides an updated comprehensive framework at the umbrella-level, integrating cardiovascular, respiratory, and safety outcomes within the same evidence system, and explicitly addressing three key issues: methodological quality, overlap of original studies, and certainty of evidence. By jointly using AMSTAR-2, GRADE, CCA, and a predefined strategy for prioritizing representative reviews, this study aims to distinguish which findings are based on higher-confidence evidence and which findings can only be considered provisional due to heterogeneity, overlap, or weaker review methods. This is particularly important in a rapidly evolving research field, as new RCT evidence may significantly alter conclusions that were initially driven primarily by observational studies.

By linking evidence certainty (GRADE), methodological rigor (AMSTAR-2), and biological plausibility within a single framework, this umbrella review provides not only a synthesis of associations but also a structured interpretive framework for understanding how immune-mediated mechanisms may contribute to cardiopulmonary outcomes across heterogeneous evidence sources.

### Strengthening the cardiovascular evidence chain

One major advantage of this umbrella review is that it integrates a stronger and methodologically more reliable cardiovascular evidence base in recent years, especially evidence from recent RCTs and RCT-focused meta-analyses. Early epidemiological studies suggested that influenza infection might trigger acute cardiovascular events, but these findings were limited by small sample sizes, high heterogeneity in study designs, as well as residual confounding and ‘healthy vaccinee bias’ (2). With the publication of the IAMI trial, as well as new quantitative synthesis studies by Liu et al. (2025) and Veroniki et al. (2024), the evidence supporting influenza vaccination as a cardiovascular prevention strategy has been significantly strengthened (4–6).

From the perspective of umbrella reviews, the most convincing signal currently comes from the reduction in MACE among patients with IHD and ACS. In the GRADE summary, the evidence for MACE in the IHD population was rated as high certainty, with a pooled relative risk of 0.67 (95% CI 0.52–0.87), based on 5 RCTs; whereas cardiovascular death in the CVD population showed a directionally consistent trend of benefit, but the certainty of evidence was relatively low (16). These findings are clinically plausible and are consistent with existing pathophysiological models, in which influenza infection can increase the risk of acute cardiovascular events through systemic inflammation, endothelial dysfunction, plaque instability, and thrombosis (20–22). However, our overall results also suggest that not all cardiovascular outcomes have the same strength of evidence. For example, although the risk of stroke showed a decreasing trend in recent meta-analyses, the certainty of evidence remains low due to very high heterogeneity and reliance on a mix of observational and randomized evidence. Therefore, the cardiovascular benefits of influenza vaccination should not be stated as having the same certain effect on all endpoints; currently, the strongest support is mainly concentrated on specific outcomes and certain high-risk populations, especially in contexts supported by recent RCT evidence.

### Mechanistic significance: why this topic is suitable for an immunology journal

The clinical associations summarized in this study are supported by a coherent immunopathophysiological framework, which also underscores the relevance of this topic to an immunology journal. Influenza infection is not merely a transient respiratory assault; it is also a systemic inflammatory event that can amplify signals of whole-body innate immune activation, cytokine release, endothelial dysfunction, platelet activation, and immunothrombosis (20–22). These processes may promote destabilization of pre-existing atherosclerotic plaques, increase myocardial oxygen demand, and facilitate thrombosis, thereby raising the likelihood of acute cardiovascular events.

From this perspective, the potential benefits of the influenza vaccine can be achieved through at least two interrelated pathways. First, by preventing or mitigating influenza infection itself, the vaccine can reduce the upstream inflammatory burden that triggers cardiopulmonary decompensation. Second, by reducing the frequency and severity of acute infections, the vaccine may interrupt subsequent immune-thrombotic cascades involving cytokine amplification, vascular activation, and plaque instability. These mechanisms may be particularly important for older adults and individuals with multiple comorbidities, as they have higher baseline inflammation, more fragile endothelium, and immunosenescence that may further increase their susceptibility to infection and cardiovascular instability. Although this umbrella review was not designed to directly assess mechanistic endpoints, the convergence of epidemiological findings, trial evidence supporting clinical benefits, and existing inflammatory biology strengthens the rationale for considering the influenza vaccine as an intervention with both anti-infective effects and indirect cardiovascular protective significance. Meanwhile, the direct immune-modulatory effects of the vaccine on vascular inflammation remain incompletely understood, and further mechanistic studies are still needed.

### Respiratory benefits in vulnerable populations

This study also emphasizes the importance of the influenza vaccine in populations at high-risk for respiratory diseases. In patients with COPD, receiving the influenza vaccine is significantly associated with a reduced risk of acute exacerbations; this outcome has clear clinical significance, as acute exacerbations are one of the key drivers of hospitalization, accelerated lung function decline, and mortality (19). In older adults, the influenza vaccine is associated with a significantly lower risk of laboratory-confirmed influenza, and this conclusion is supported by high-certainty evidence based on meta-analyses of RCTs (12).

The significance of these findings is not limited to respiratory medicine but should be understood within a broader cardio-pulmonary interaction framework. There is a bidirectional relationship between respiratory infections and cardiovascular decompensation: lung infections can trigger systemic inflammation and hemodynamic stress, while cardiovascular disease can reduce physiological reserve, making patients more susceptible to adverse outcomes following respiratory infections. Therefore, reducing respiratory morbidity related to influenza may also have downstream significance for cardiovascular stability, especially in patients with cumulative chronic disease burden. However, these respiratory benefits should still be interpreted with caution. Certain outcomes, such as acute COPD exacerbations, show an overall favorable trend, but due to significant heterogeneity among the included studies, the certainty of evidence is only low. This means that the direction of ‘possible benefit’ is relatively clear, but the true magnitude of the effect remains uncertain.

### Safety and clinical acceptability

Safety remains one of the most important factors determining vaccination rates in clinical practice. In the evidence base included in this study, based on trial-level data, influenza vaccines were not associated with a clear increase in serious adverse events or other major safety issues (16). In the GRADE summary, the evidence for serious adverse events in the CVD population was rated as moderate certainty, and no statistically significant increase in risk was observed compared with the control group. This is particularly important for high-risk populations, as in these groups, doctors and patients often have reservations about vaccination due to concerns about frailty, complex comorbidities, or treatment burden. Overall, the existing evidence supports a favorable risk–benefit profile for influenza vaccines in vulnerable populations, but safety interpretations should still be based on specific outcomes and existing evidence, rather than overgeneralized.

### Interpret the results from the perspective of methodological quality and certainty of evidence

A key objective of this umbrella review is not simply to summarize the effect sizes already published, but to further determine: for different parts of the evidence system, how confident can we truly be. This is very important because there are significant differences in methodological quality among the included reviews. Some reviews are rated as high or moderate quality, while others are rated as low or critically low quality in AMSTAR-2, common reasons including lack of protocol registration, insufficient exploration of bias, or incomplete reporting of funding sources and search methods. Therefore, when interpreting the results, we deliberately stratify based on both the methodological quality of the reviews and the certainty of the evidence according to GRADE.

The most reliable conclusions in this study mainly come from recent evidence based on RCTs with high certainty ratings for outcomes, such as the reduction of MACE in the IHD population and the prevention of laboratory-confirmed influenza in older adults. In contrast, outcomes primarily supported by observational studies, mixed designs, or highly heterogeneous meta-analyses, such as stroke incidence and certain respiratory and vascular adverse event endpoints, should be interpreted more cautiously. It is necessary to emphasize this because an umbrella review that indiscriminately aggregates different outcomes can give the impression that ‘all results are of similar strength,’ whereas in reality, the underlying evidence structure is not balanced. Our use of AMSTAR-2 and GRADE is precisely to avoid such overinterpretation and to clarify which conclusions can be accepted with high confidence and which remain at a moderate or limited confidence level.

### Methodological considerations and handling of overlaps

This study has several methodological advantages. First, we explicitly assessed the overlap of primary studies among different reviews using a citation matrix and CCA, rather than treating all meta-analyses as independent sources of evidence. The degree of overlap varied from very slight to moderate across different outcome domains, suggesting that some redundancy does exist, but not enough to fully explain the main patterns at the umbrella-level. Second, we used predefined priority rules to identify representative reviews when outcomes overlapped, giving higher interpretive weight to more recent, reliable, and RCT-focused syntheses. Third, we did not perform new quantitative pooling across different overlapping reviews, thereby minimizing the risk of double-counting the same underlying primary studies. Therefore, this study is better understood as a structured, umbrella-level narrative synthesis, with interpretations based on methodological appraisal, certainty of evidence, and overlap analysis.

We have also introduced an automated assisted screening process to improve the efficiency and reproducibility of the early literature identification stage. It is important to emphasize that this system does not replace human judgment. Automated tools are only used to support preliminary prioritization and relevance assessment, while all final inclusion and exclusion decisions are made by human researchers. Therefore, this process is consistent with the emerging principles for transparent and auditable AI-assisted evidence synthesis, while retaining the necessary human review oversight in traditional systematic reviews.

### Limitations

This study also has several limitations. First, the conclusions of an umbrella review are inevitably constrained by the quality of the included reviews; when the underlying reviews have weak methodologies, our confidence in the corresponding conclusions naturally decreases. Second, some clinically important subgroups still lack sufficient evidence, including patients with heart failure, atrial fibrillation, chronic kidney disease, and multimorbidity. Third, several outcomes still show significant heterogeneity, especially stroke and certain respiratory endpoints, which limits the precision and generalizability of the pooled estimates. Fourth, although we have formally assessed and managed study overlap, conceptual overlap among related outcomes and similar populations at the umbrella review level cannot be completely eliminated. Finally, although the mechanistic framework proposed in this paper is biologically plausible, this study synthesizes evidence based on clinical outcomes rather than directly on mechanistic biomarker data; therefore, the related causal pathways should be considered supportive interpretations rather than definitively confirmed mechanisms.

### Clinical significance

Overall, the findings of this study support the use of the influenza vaccine as an important component of preventive management for individuals at high cardiopulmonary risk. Currently, the strongest evidence supports its inclusion in cardiovascular secondary prevention strategies for specific high-risk groups, while respiratory and safety outcomes further reinforce its value as a broadly applicable preventive intervention. Given that the influenza vaccine is relatively low in cost, highly scalable, and already recommended by multiple major professional societies, increasing vaccination rates should still be regarded as a realistic and important public health priority. Strategies such as vaccination pathways at discharge, electronic medical record reminders, integration into cardiac rehabilitation and chronic disease management programs, and coordinated outpatient follow-up may all be feasible ways to narrow the persistent evidence-practice gap.

### Future research directions

Although the evidence base is strengthening, several important gaps remain. Future research should prioritize:

1. Conducting large-sample RCTs in cardiovascular subgroups with insufficient evidence, including heart failure with preserved ejection fraction, atrial fibrillation, chronic kidney disease, and populations with multimorbidity;
2. Conducting comparative effectiveness studies across different vaccine platforms and formulations, including high-dose, adjuvanted, recombinant vaccines, and emerging technologies;
3. Conducting mechanistic studies focusing on immune, vascular, thrombotic, and inflammatory pathways, especially in the context of aging and immunosenescence;
4. Evaluating the cumulative benefits and potential non-influenza season protective effects of consecutive annual vaccinations through longitudinal studies;
5. Conducting implementation and health system research to identify the most effective strategies to increase vaccination rates in high-risk populations.

## Conclusion

**Figure 5:**
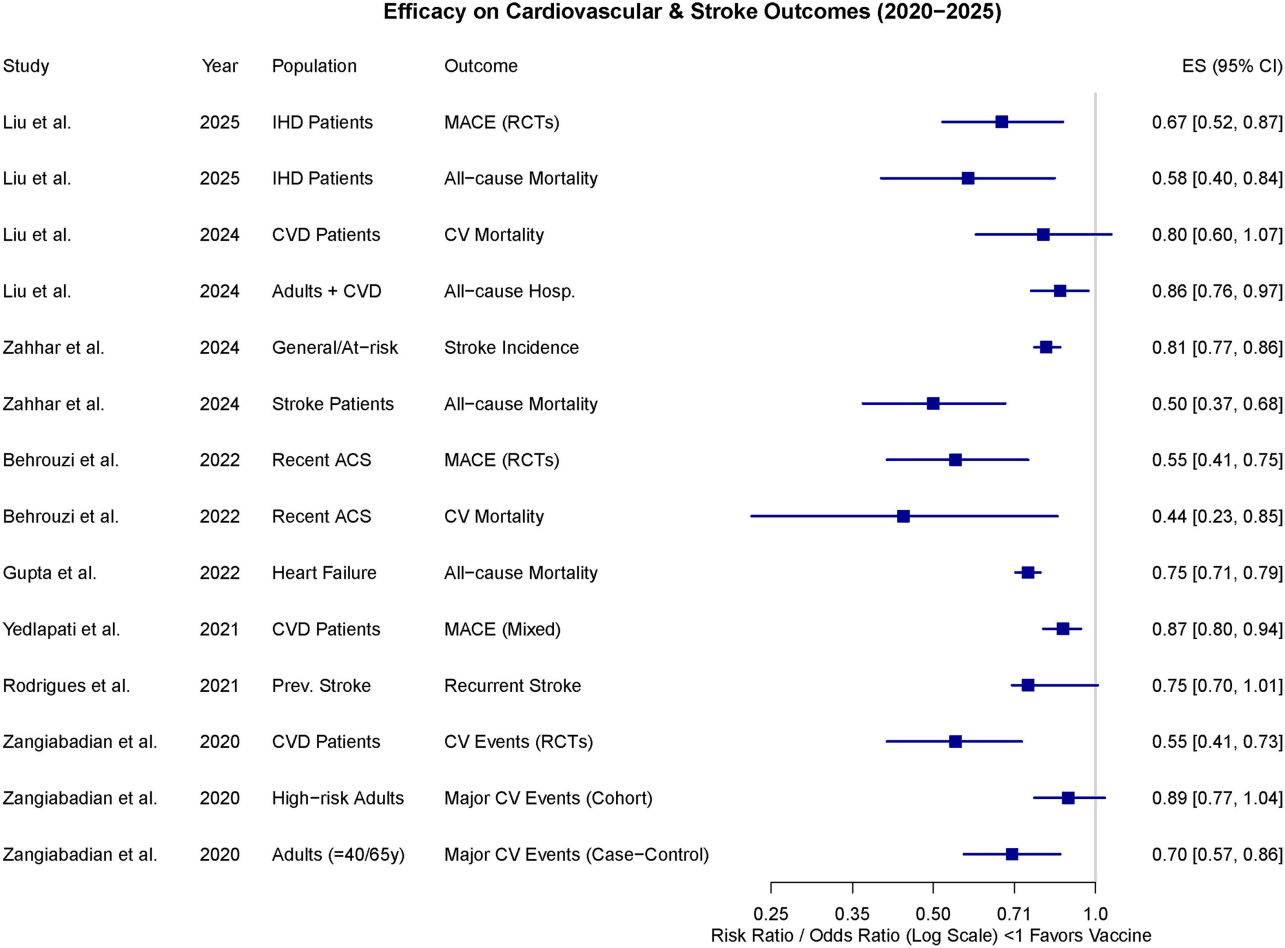
PRISMA 2020 flow diagram of study selection. A total of 1,072 records were identified from PubMed via the Entrez API (n = 363), the Cochrane Library (n = 676), and Embase (n = 393). After removal of 264 duplicates and 182 records marked as ineligible by automation tools, 808 records were screened and 354 were excluded. Of 454 reports sought for retrieval, 86 were not retrieved, leaving 368 reports for full-text assessment. After full-text review, 354 reports were excluded because of wrong population (n = 155), wrong outcome (n = 86), non-target disease population (n = 47), or statistical errors (n = 66). Fourteen studies were ultimately included in the umbrella review.

The influenza vaccine appears to be a safe and potentially important preventive strategy, associated with reduced cardiovascular and respiratory morbidity in high-risk populations, with the strongest current evidence mainly supporting certain cardiovascular endpoints and influenza-related outcomes. The incremental contribution of this umbrella review to the existing literature lies in integrating updated evidence across outcomes while explicitly addressing the methodological quality of reviews, overlap of original studies, and certainty of evidence. These findings support incorporating the influenza vaccine into routine preventive care for vulnerable cardiopulmonary populations and also indicate that further mechanistic, subgroup, and implementation studies are still needed. The proposed immunological and pathophysiological mechanisms are shown in **Figure 5**.
