# Supplementary material for: Influenza vaccination and cardiovascular and respiratory outcomes in high-risk populations: an umbrella review of systematic reviews and meta-analyzes
Source: Front Immunol. 2026 May 26;17:1798398. doi: 10.3389/fimmu.2026.1798398 (PMC13246626; doi:10.3389/fimmu.2026.1798398)
Supplement: Supplementary file 1 [file DataSheet1.docx]

**Supplementary Appendix 2. Automation-Assisted Screening Workflow and System Architecture**

This appendix provides a detailed description of the automation-assisted literature screening workflow used in this umbrella review. The workflow is implemented through a custom-built system designed to improve efficiency during initial PubMed screening while maintaining full human oversight. The system integrates PubMed Entrez APIs, full-text prioritization modules, and large-language-model (LLM)–assisted relevance assessment. All final inclusion or exclusion decisions were made by human reviewers.

**1. System Overview and Architecture**

The automated screening system consists of four major components.

**1.1 PubMed API Integration**

The system uses the NCBI Entrez Programming Utilities (E-utilities) to perform:

- Paginated PubMed searches
- Retrieval of metadata (title, abstract, journal, publication year)
- Query expansion and optimized retry mechanisms

A unified TLS-certification handler ensures secure API communication across different execution environments.

**1.2 Full-Text Retrieval Pipeline**

To maximize evaluation accuracy, the workflow incorporates a **“full-text first”** strategy:

1. **PMCID → JATS XML retrieval**
   For records available in PubMed Central, full JATS XML is retrieved and parsed (using *BeautifulSoup*) to extract structured sections (title, abstract, methods, results, discussion, full body).
2. **DOI-based HTML fallback**
   For records without a PMCID, the system attempts to fetch the DOI-resolved web version of the article.
3. **Abstract-only fallback**
   If neither JATS nor DOI full text is accessible, the system uses the abstract (only when strict full-text mode is not enforced).

This step mirrors emerging PRISMA-AI recommendations for transparency and reproducibility in automation-assisted screening.

**1.3 Large-Language-Model (LLM) Relevance Evaluation**

The system calls **ChatGPT-4o** through an API interface. Key design features include:

**Prompt structure**
Each article is evaluated using a structured prompt containing:

- Title
- Abstract
- Methods
- Results
- Discussion
- Full body text (if available)
- The target research question

The model returns content in a structured JSON format, including:

- is_topic_match (Boolean)
- confidence (0–1 score)
- key_matches
- key_mismatches
- quotes (short excerpts used for decision justification)
- verdict_reason

**Five-round independent evaluation**
To ensure robustness:

- Each article undergoes **five independent** LLM inference rounds.
- The system selects the decision with the **highest confidence score**.

This mitigates LLM variability and increases reproducibility.

**1.4 Human Oversight and Decision Governance**

Although the system automates early steps, human reviewers make all final decisions:

- Articles marked as “potentially relevant” are manually reviewed by two independent reviewers.
- Disagreements are resolved by consensus or adjudication by a senior reviewer.
- Articles excluded by AI are manually checked when classification uncertainty exists.

The automated system accelerates screening but does **not** replace human judgment.

**2. Search-Term Generation Module**

To accommodate topics with evolving terminology, the system offers two modes:

1. **AI-generated search mode**
   - The user inputs a research topic title.
   - ChatGPT-4o generates a high-recall PubMed Boolean query.
   - Optional sanitization removes nonsensical or overly narrow terms.
2. **Deterministic mode**
   - The user directly inputs a manually constructed PubMed query.

The system includes fallback mechanisms that modify overly restrictive queries (e.g., removing nested parentheses or keeping only essential keywords) to avoid zero-result failures.

**3. Article Screening Pipeline**

The screening workflow proceeds through the following steps.

**Step 1: Identification**

- Perform PubMed search with paginated retrieval.
- Apply fallback expansion if no results are initially found.

**Step 2: Full-Text Prioritization**

- Attempt PMCID → JATS XML retrieval.
- If unavailable, retrieve DOI-based HTML full text.
- If still unavailable, use abstract-only evaluation.

**Step 3: LLM Evaluation**

- Create the prompt dynamically from extracted article sections.
- Run five inference cycles.
- Select the highest-confidence decision.

**Step 4: Logging and Evidence Tracking**
For each screened article, the system logs:

- Raw decision output
- Key textual evidence used by the LLM
- Inclusion/exclusion rationale
- A 2–3 sentence article summary generated separately

**Step 5: Human Review**

- Humans validate all AI-flagged inclusions.
- AI exclusions are revisited when ambiguous.

**Step 6: Output**
A structured CSV file is generated containing:

- Title
- First author
- Publication year
- Journal
- Full-text source
- Summary
- Inclusion status
- Inclusion/exclusion rationale

This file forms the audit trail required for reproducible review methodology.

**4. System Safeguards**

The system includes several built-in safety features.

**4.1 Hard Deadlines**

Each article evaluation has a configurable timeout (default: 120 seconds).

**4.2 Graceful Stop Mechanism**

A global stop_event allows the user to halt the entire workflow at safe checkpoints.

**4.3 Retry Logic**

Robust retry loops are implemented for:

- API failures
- Network timeouts
- PMC or DOI retrieval errors

**4.4 TLS and Certificate Handling**

Automatic detection of enterprise or *certifi* certificate bundles ensures secure communication.

**5. Reproducibility and Transparency Considerations**

The automation-assisted screening system follows these principles:

- All AI decisions are auditable and stored with explicit reasoning and quoted evidence.
- No inclusion/exclusion decision is made automatically, maintaining adherence to PRISMA and Cochrane standards.
- Full-text prioritization enhances the accuracy of LLM-based relevance assessment.
- Fallback strategies prevent data loss due to unavailable full texts.
- Screening decisions are reproducible within the defined stochastic evaluation rounds, enabling methodological audits.

**6. Limitations**

- Full automation is not feasible due to legal, technical, and methodological constraints; human oversight remains essential.
- DOI-based full-text retrieval may fail depending on publisher restrictions.
- LLM performance depends on prompt design and model updates; versioning is logged accordingly.

**Conclusion**

The automation-assisted workflow described here provides a flexible, transparent, and reproducible system for large-scale literature screening in umbrella reviews. By integrating API-based retrieval, full-text prioritization, structured LLM evaluation, and human oversight, the system enhances efficiency while preserving methodological rigor.
