# Supplementary figures and images for: Influenza vaccination and cardiovascular and respiratory outcomes in high-risk populations: an umbrella review of systematic reviews and meta-analyzes
Source: Front Immunol. 2026 May 26;17:1798398. doi: 10.3389/fimmu.2026.1798398 (PMC13246626; doi:10.3389/fimmu.2026.1798398)

AMSTAR 2 Quality Assessment Heatmap

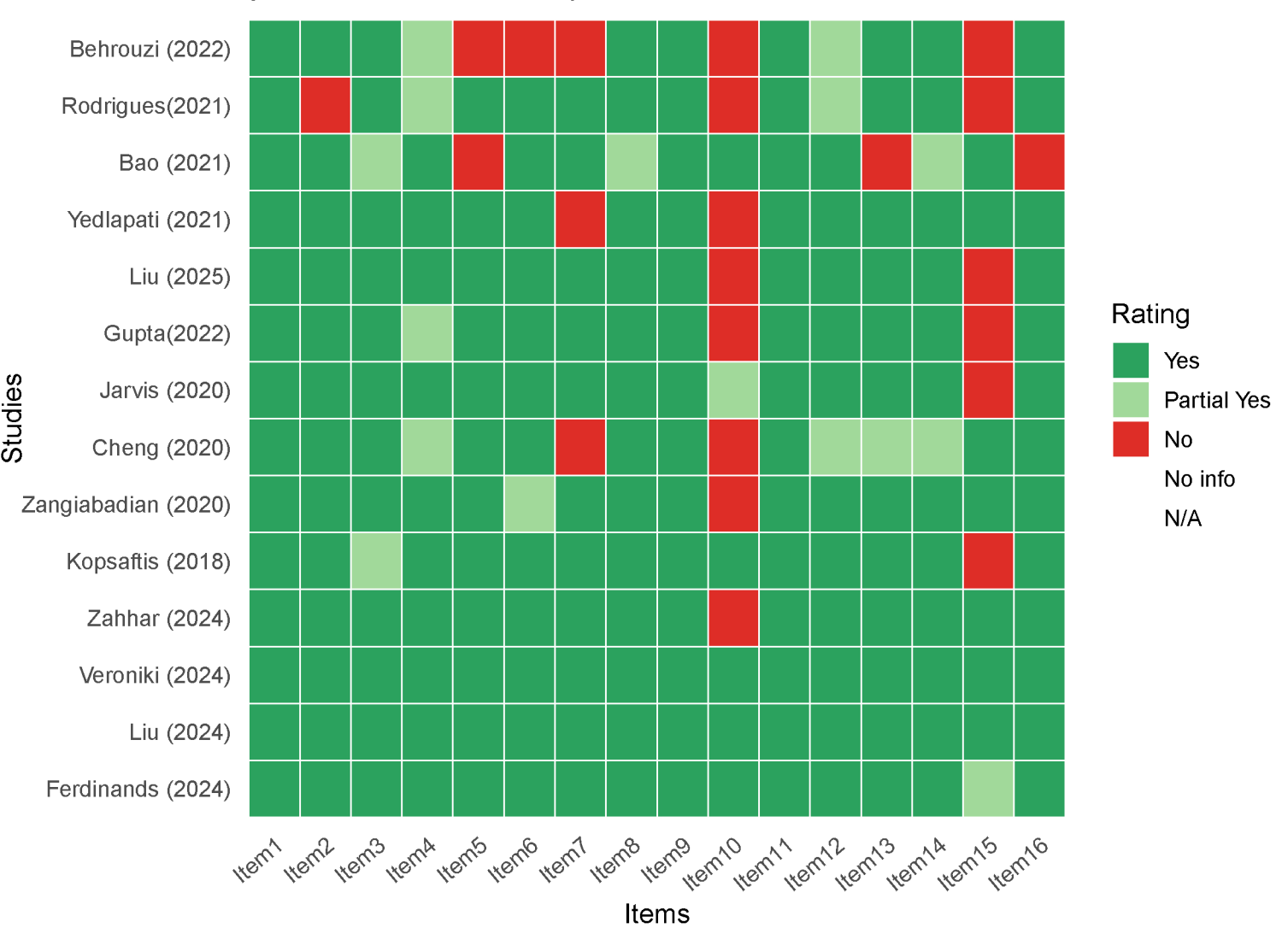

Supplement: Supplementary file 2 [file Image1.pdf]

AMSTAR 2 Overall Quality Levels

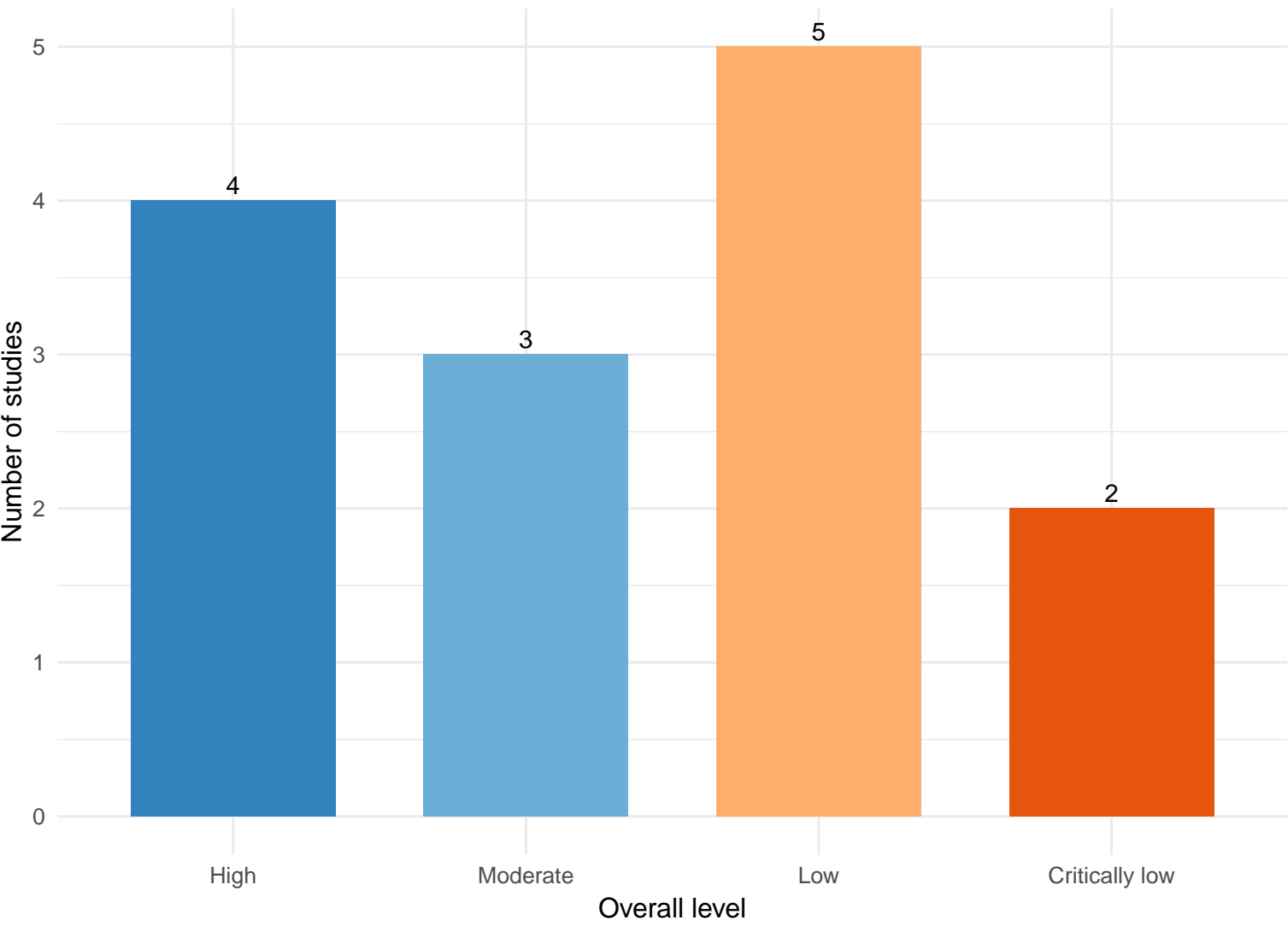

Supplement: Supplementary file 3 [file Image2.pdf]
