# Supplementary material for: Influenza vaccination and cardiovascular and respiratory outcomes in high-risk populations: an umbrella review of systematic reviews and meta-analyzes
Source: Front Immunol. 2026 May 26;17:1798398. doi: 10.3389/fimmu.2026.1798398 (PMC13246626; doi:10.3389/fimmu.2026.1798398)
Supplement: Supplementary file 4 [file Image3.pdf]

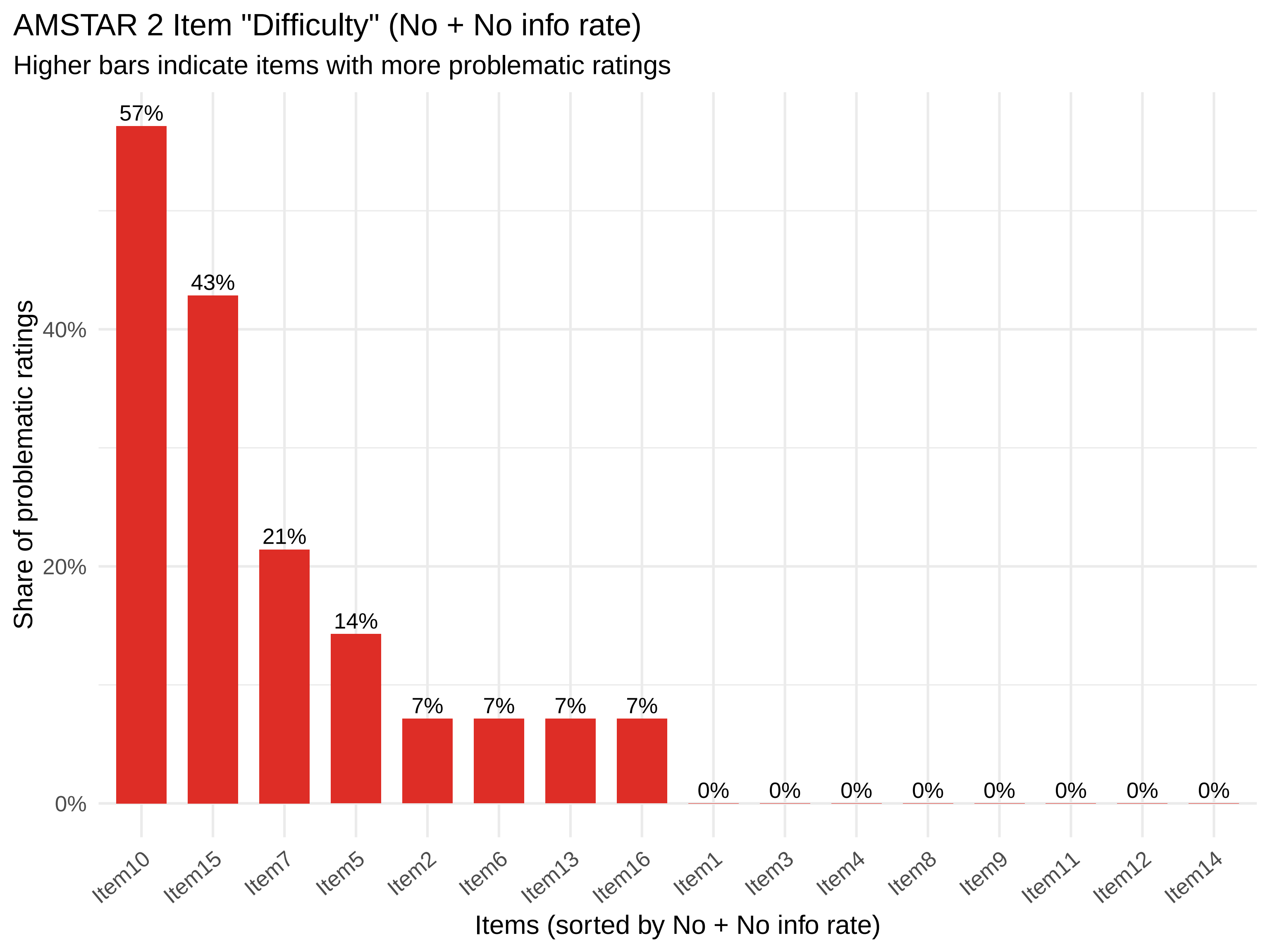
AMSTAR 2 Item "Difficulty" (No + No info rate)
Higher bars indicate items with more problematic ratings
Share of problematic ratings
0%
20%
40%
57%
43%
21%
14%
7%
7%
7%
7%
0%
0%
0%
0%
0%
0%
0%
0%
Item10
Item15
Item7
Item5
Item2
Item6
Item13
Item16
Item1
Item3
Item4
Item8
Item9
Item11
Item12
Item14
Items (sorted by No + No info rate)
