# Supplementary material for: Influenza vaccination and cardiovascular and respiratory outcomes in high-risk populations: an umbrella review of systematic reviews and meta-analyzes
Source: Front Immunol. 2026 May 26;17:1798398. doi: 10.3389/fimmu.2026.1798398 (PMC13246626; doi:10.3389/fimmu.2026.1798398)
Supplement: Supplementary file 6 [file Image5.pdf]

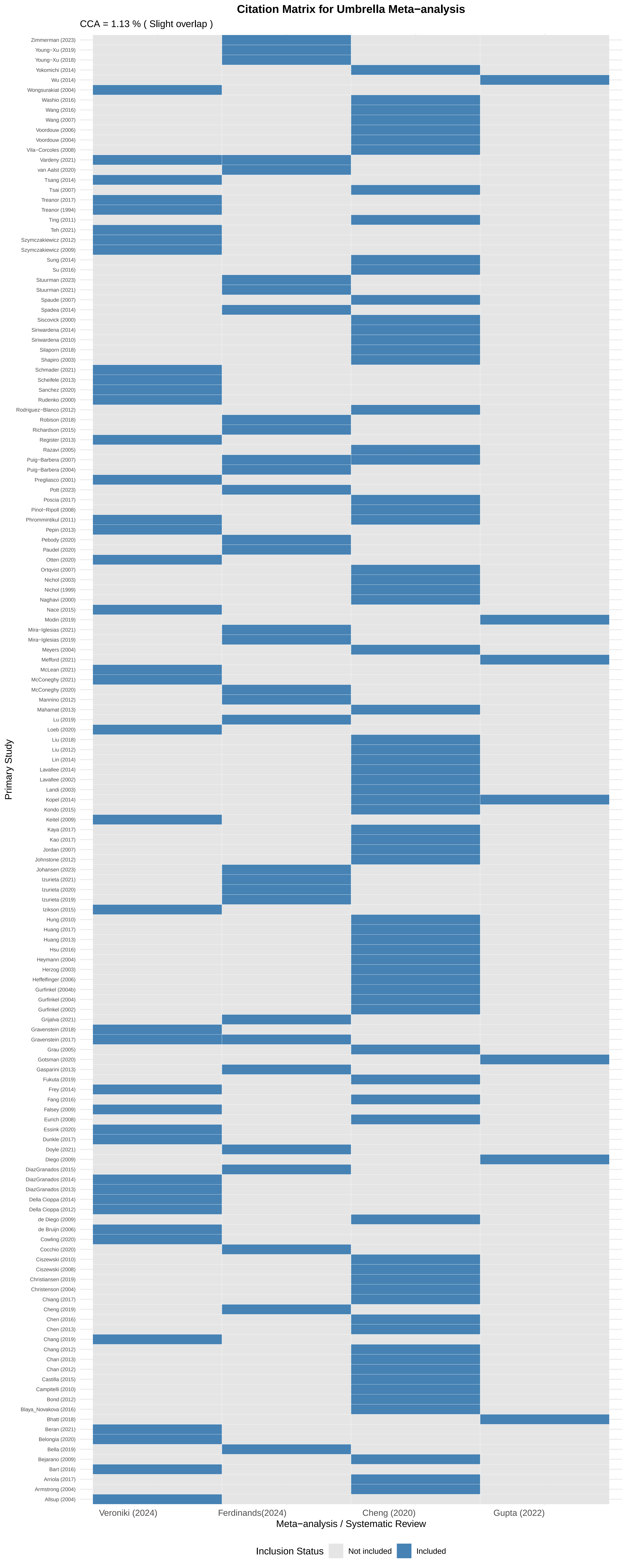

Citation Matrix for Umbrella Meta−analysis
CCA = 1.13 % (Slight overlap)
Primary Study
Zimmerman (2023)
Young−Xu (2019)
Young−Xu (2018)
Yokomichi (2014)
Wu (2014)
Wongsurakiat (2004)
Washio (2016)
Wang (2016)
Wang (2007)
Voordouw (2006)
Voordouw (2004)
Vila−Corcoles (2008)
Vardeny (2021)
van Aalst (2020)
Tsang (2014)
Tsai (2007)
Treanor (2017)
Treanor (1994)
Ting (2011)
Teh (2021)
Szymczakiewicz (2012)
Szymczakiewicz (2009)
Sung (2014)
Su (2016)
Stuurman (2023)
Stuurman (2021)
Spaude (2007)
Spadea (2014)
Siscovick (2000)
Siriwardena (2014)
Siriwardena (2010)
Silaporn (2018)
Shapiro (2003)
Schmader (2021)
Scheifele (2013)
Sanchez (2020)
Rudenko (2000)
Rodriguez−Blanco (2012)
Robison (2018)
Richardson (2015)
Register (2013)
Razavi (2005)
Puig−Barbera (2007)
Puig−Barbera (2004)
Pregliasco (2001)
Pott (2023)
Poscia (2017)
Pinol−Ripoll (2008)
Phrommintikul (2011)
Pepin (2013)
Pebody (2020)
Paudel (2020)
Otten (2020)
Ortqvist (2007)
Nichol (2003)
Nichol (1999)
Naghavi (2000)
Nace (2015)
Modin (2019)
Mira−Iglesias (2021)
Mira−Iglesias (2019)
Meyers (2004)
Mefford (2021)
McLean (2021)
McConeghy (2021)
McConeghy (2020)
Mannino (2012)
Mahamat (2013)
Lu (2019)
Loeb (2020)
Liu (2018)
Liu (2012)
Lin (2014)
Lavallee (2014)
Lavallee (2002)
Landi (2003)
Kopel (2014)
Kondo (2015)
Keitel (2009)
Kaya (2017)
Kao (2017)
Jordan (2007)
Johnstone (2012)
Johansen (2023)
Izurieta (2021)
Izurieta (2020)
Izurieta (2019)
Izikson (2015)
Hung (2010)
Huang (2017)
Huang (2013)
Hsu (2016)
Heymann (2004)
Herzog (2003)
Heffelfinger (2006)
Gurfinkel (2004b)
Gurfinkel (2004)
Gurfinkel (2002)
Grijalva (2021)
Gravenstein (2018)
Gravenstein (2017)
Grau (2005)
Gotsman (2020)
Gasparini (2013)
Fukuta (2019)
Frey (2014)
Fang (2016)
Falsey (2009)
Eurich (2008)
Essink (2020)
Dunkle (2017)
Doyle (2021)
Diego (2009)
DiazGranados (2015)
DiazGranados (2014)
DiazGranados (2013)
Della Cioppa (2014)
Della Cioppa (2012)
de Diego (2009)
de Bruijn (2006)
Cowling (2020)
Cocchio (2020)
Ciszewski (2010)
Ciszewski (2008)
Christiansen (2019)
Christenson (2004)
Chiang (2017)
Cheng (2019)
Chen (2016)
Chen (2013)
Chang (2019)
Chang (2012)
Chan (2013)
Chan (2012)
Castilla (2015)
Campitelli (2010)
Bond (2012)
Blaya_Novakova (2016)
Bhatt (2018)
Beran (2021)
Belongia (2020)
Bella (2019)
Bejarano (2009)
Bart (2016)
Arriola (2017)
Armstrong (2004)
Allsup (2004)
Veroniki (2024)
Ferdinands(2024)
Cheng (2020)
Gupta (2022)
Meta−analysis / Systematic Review
Inclusion Status
Not included
Included
