# Supplementary material for: Influenza vaccination and cardiovascular and respiratory outcomes in high-risk populations: an umbrella review of systematic reviews and meta-analyzes
Source: Front Immunol. 2026 May 26;17:1798398. doi: 10.3389/fimmu.2026.1798398 (PMC13246626; doi:10.3389/fimmu.2026.1798398)
Supplement: Supplementary file 8 [file Table1.docx]

**Supplementary Table S1. Selection of Primary Systematic Reviews for Overlapping Outcomes**

| **Outcome Domain** | | **Overlapping Reviews Identified** | | **Selection Criteria Applied** | | **Selected Representative Review** | | **Justification for Selection** | |
| --- | --- | --- | --- | --- | --- | --- | --- | --- | --- |
| Cardiovascular: MACE | | Liu (2025), Behrouzi (2022), Zangiabadian (2020), Yedlapati (2021) | | 1. Recency of search; 2. Focus on RCTs | | Liu et al. (2025) | | Most recent publication (2025); synthesized exclusively RCT data for IHD patients; provides the most up-to-date evidence on major adverse cardiovascular events. | |
| Cardiovascular: Mortality | | Liu (2025), Liu (2024), Behrouzi (2022), Gupta (2022) | | 1. Population specificity; 2. Focus on RCTs | | Liu et al. (2025) & Liu et al. (2024) | | Liu (2025) provides data on all-cause mortality in IHD patients. Liu (2024) offers specific data on cardiovascular mortality in CVD patients. Both were retained to cover different mortality endpoints comprehensively. | |
| Cardiovascular: CV Events | | Zangiabadian (2020), Yedlapati (2021) | | 1. Study design variety; 2. Sample size | | Zangiabadian et al. (2020) | | Selected for its unique contribution of stratified data across three study designs (RCT, Cohort, Case-Control), allowing for a robust comparison of evidence levels. | |
| Cardiovascular: Stroke | | Zahhar (2024), Rodrigues (2021) | | 1. Sample size; 2. Recency | | Zahhar et al. (2024) | | Overwhelmingly larger sample size (>200 million); updated comprehensive search including both stroke incidence and mortality in general/at-risk populations. | |
| Respiratory: COPD Exacerbation | | Bao (2021), Kopsaftis (2018), Cheng (2020) | | 1. Number of primary studies; 2. Recency | | Bao et al. (2021) | | Included the largest number of relevant studies (k=10); most recent update for this specific outcome with a focus on acute exacerbations. | |
| Elderly: Influenza Infection | | Veroniki (2024), Cheng (2020), Ferdinands (2024) | | 1. Methodological quality (AMSTAR-2); 2. Study design (RCT) | | Veroniki et al. (2024) | | Rated "High" on AMSTAR-2; synthesized high-quality RCT data via Network Meta-Analysis; represents the highest level of evidence for efficacy in older adults. | |
| Elderly: Hospitalization | | Veroniki (2024), Ferdinands (2024) | | 1. Real-world relevance; 2. Specific outcome definition | | Ferdinands et al. (2024) & Veroniki et al. (2024) | | Both were retained to provide complementary perspectives: Veroniki for RCT data (efficacy) and Ferdinands for observational data (real-world effectiveness). | |
| Safety: Adverse Events | | Liu (2024), Veroniki (2024) | | 1. Population match; 2. Outcome specificity | | Liu et al. (2024) | | Selected as the primary source for safety data in patients with CVD because it provides outcome-specific reporting of serious adverse events based on updated RCT evidence. | |
| Outcome Domain | Overlapping Reviews Identified | | Selection Criteria Applied | | Selected Representative Review | | Justification for Selection | |  |
