# Supplementary material for: Influenza vaccination and cardiovascular and respiratory outcomes in high-risk populations: an umbrella review of systematic reviews and meta-analyzes
Source: Front Immunol. 2026 May 26;17:1798398. doi: 10.3389/fimmu.2026.1798398 (PMC13246626; doi:10.3389/fimmu.2026.1798398)
Supplement: Supplementary file 9 [file Table2.docx]

**Supplementary Table S2. GRADE Summary of Findings for Influenza Vaccination Outcomes**

| **Outcome** | **Relative Effect (95% CI)** | **No of Participants (Studies)** | **Certainty of Evidence (GRADE)** | **Comments** |
| --- | --- | --- | --- | --- |
| **Cardiovascular Protection** |  |  |  |  |
| 1. MACE in IHD(Liu et al. 2025) | RR 0.67(0.52 to 0.87) | 4,656(5 RCTs) | ⊕⊕⊕⊕High | No serious limitations. Although the review quality was rated Low on AMSTAR-2, the included primary RCTs were rigorous with low heterogeneity ($I^2=36%$), increasing confidence in the estimate. |
| 2. CV Mortality(Liu et al. 2024) | RR 0.80(0.60 to 1.07) | 8,358(3 RCTs) | ⊕⊕⊕◯Moderate | Imprecision: Downgraded once because the confidence interval crosses the line of no effect (1.0), indicating uncertainty about the benefit magnitude. |
| 3. Stroke Incidence(Zahhar et al. 2024) | OR 0.81(0.77 to 0.86) | >200M(44 Studies) | ⊕⊕◯◯Low | Inconsistency: Downgraded twice due to extreme heterogeneity ($I^2=98%$) unexplained by subgroup analysis. Evidence comes from mixed designs (Obs + RCTs). |
| **Respiratory & Vulnerable** |  |  |  |  |
| 4. COPD Exacerbation(Bao et al. 2021) | RR 0.37(0.21 to 0.61) | 17,972(10 Studies) | ⊕⊕◯◯Low | Inconsistency: Downgraded due to very high heterogeneity ($I^2=98%$). Risk of Bias: Review rated "Low" on AMSTAR-2. |
| 5. Flu Infection (Older Adults)(Veroniki et al. 2024) | OR 0.23(0.11 to 0.51) | 52,202(9 RCTs) | ⊕⊕⊕⊕High | High Quality. Based on high-quality RCTs with zero heterogeneity ($I^2=0%$). Strongest evidence for vaccine efficacy in this population. |
| 6. ILI (Older Adults)(Veroniki et al. 2024) | OR 0.39(0.15 to 1.02) | 854(2 RCTs) | ⊕⊕◯◯Low | Imprecision: Downgraded twice due to small sample size (N<1,000) and wide CI crossing the null effect line. |
| **Safety** |  |  |  |  |
| 7. Serious Adverse Events (SAE)(Liu et al. 2024) | RR 1.14(0.73 to 1.77) | 3,229(2 RCTs) | ⊕⊕⊕◯Moderate | Imprecision: Downgraded once due to wide confidence interval crossing 1.0. However, the result indicates no statistically significant increase in harm compared to control. |
| 8. Vascular AEs (High-Dose)(Veroniki et al. 2024) | IRR 0.69(0.49 to 0.97) | ~50,000(4 RCTs) | ⊕⊕◯◯Low | Inconsistency: Downgraded twice due to very high heterogeneity ($I^2=97%$) among the included trials. |

**Note:**MACE: Major Adverse Cardiovascular Events; CV: Cardiovascular; IHD: Ischemic Heart Disease; ILI: Influenza-like Illness; AEs: Adverse Events; RR: Risk Ratio; OR: Odds Ratio; IRR: Incidence Rate Ratio; CI: Confidence Interval.

GRADE Working Group grades of evidence:

High certainty (⊕⊕⊕⊕): We are very confident that the true effect lies close to that of the estimate of the effect.

Moderate certainty (⊕⊕⊕◯): We are moderately confident in the effect estimate: The true effect is likely to be close to the estimate of the effect, but there is a possibility that it is substantially different.

Low certainty (⊕⊕◯◯): Our confidence in the effect estimate is limited: The true effect may be substantially different from the estimate of the effect.

Very low certainty (⊕◯◯◯): We have very little confidence in the effect estimate: The true effect is likely to be substantially different from the estimate of effect.
