# Supplementary material for: Influenza vaccination and cardiovascular and respiratory outcomes in high-risk populations: an umbrella review of systematic reviews and meta-analyzes
Source: Front Immunol. 2026 May 26;17:1798398. doi: 10.3389/fimmu.2026.1798398 (PMC13246626; doi:10.3389/fimmu.2026.1798398)
Supplement: Supplementary file 10 [file Table3.docx]

**Supplementary Figures S1–S6 — Figure Legends**

**Supplementary Figure S1. AMSTAR-2 quality assessment heatmap of all included systematic reviews and meta-analyses.**

This heatmap displays item-level ratings (“Yes,” “Partial Yes,” “No,” and “No information”) for each included review across the 16 AMSTAR-2 domains. Green shading indicates adequate methodological performance, yellow denotes partial fulfillment, and red signifies critical methodological deficiencies. The plot provides a visual overview of between-study variability in methodological rigor.

**Supplementary Figure S2. Distribution of overall AMSTAR-2 quality ratings across included reviews.**

This bar chart summarizes the final overall methodological quality classification (“High,” “Moderate,” “Low,” and “Critically low”) for all included systematic reviews and meta-analyses. The distribution reflects substantial heterogeneity in review quality, with only a minority meeting high-quality methodological standards.

**Supplementary Figure S3. AMSTAR-2 item difficulty ranked by the proportion of problematic ratings (No + No information).**

This figure ranks all AMSTAR-2 items based on the percentage of reviews receiving problematic ratings. Higher bars indicate domains where methodological deficiencies were most common, highlighting frequently unmet criteria such as protocol registration, consideration of risk of bias, and reporting of funding sources for included studies.

**Supplementary Figure S4. Citation matrix and corrected covered area (CCA) for reviews addressing respiratory outcomes and vulnerable populations.**

This matrix illustrates the overlap of primary studies across included reviews within the respiratory outcomes domain. Blue cells indicate inclusion of a primary study in a given review. The calculated CCA was 4.55%, indicating *slight* overlap, suggesting minimal redundancy and a low risk of bias due to duplicated evidence.

**Supplementary Figure S5. Citation matrix and corrected covered area (CCA) for reviews addressing cardiovascular outcomes.**

This matrix summarizes cross-review inclusion of primary cardiovascular studies. The CCA was 1.13%, reflecting *very slight* overlap, meaning the cardiovascular evidence base was largely independent across systematic reviews, enhancing the robustness of synthesized conclusions.

**Supplementary Figure S6. Citation matrix and corrected covered area (CCA) for safety outcomes and mixed clinical outcomes.**

This matrix presents study overlap for reviews evaluating safety endpoints and comprehensive clinical outcomes. A CCA of 9.72% was observed, representing *moderate overlap*. Although higher than in other domains, the overlap remains acceptable and does not materially compromise evidence independence.
